# Indications for the removal of implants after fracture healing: A comparison between human and veterinary medicine

**DOI:** 10.17221/52/2023-VETMED

**Published:** 2023-07-27

**Authors:** Mario Candela Andrade, Ignacio De Rus Aznar, Mathias Brunnberg, Pavel Slunsky

**Affiliations:** ^1^Department of Human Anatomy, Health and Medical University Potsdam, Potsdam, Brandenburg, Germany; ^2^Orthopaedic Surgery and Traumatology, University Hospital of Torrejón, Madrid, Spain; Shoulder and Knee Surgery Department, Olympia Quironsalud Hospital, Madrid, Spain; ^3^Surgical Department, Small Animal Clinic, Tierarztpraxis Sörensen, Berlin, Germany; ^4^Surgical Department, Small Animal Hospital, Anicura Kleintierspezialisten Augsburg, Augsburg, Bavaria, Germany

**Keywords:** cat, dog, explants, hardware removal, horse, metal implant

## Abstract

Indications for implant removal after fracture healing are still under debate in both human and veterinary medicine. Although hardware removal is a common procedure, it should not be undertaken lightly. Intra and post-operative complications are common and a thorough evaluation of the risks and benefits should be performed. This review aimed to collect and summarise published data on the indications for implant removal in small animals, compare the collected data with human and equine medicine, and investigate the existence of guidelines for this purpose. There is no international consensual agreement for implant removal after fracture healing, neither in small animals nor in human orthopaedics. Decision-making processes are still controversial in some scenarios, thus clear evidence-based protocols for implant removal are needed.

## INTRODUCTION

Indications for implant removal after treatment of fractures are not well defined in human orthopaedics and so far, Germany is the only country with a clear guideline for this purpose (Vos and Verhofstad 2013; DGU 2018). Small animal orthopaedics has developed enormously during the last few years. Recommendations in humans state that the removal of implants should not be performed routinely, but only in cases with clear indications such as implant-associated pain, implant failure, metal allergies, risk of periprosthetic fractures, functional limitations, or infection (Richards et al. 1992; Muller-Farber 2003; Townend and Parker 2005; Vos et al. 2012; Acklin et al. 2018; Jain et al. 2019).

In small animal orthopaedics, implant removal is mostly performed when complications, such as loosened or broken implants, corrosion, interference with bone growth, soft tissue irritation, or infection, are present. Elective surgery is only carried out in 6% of the cases (Emmerson and Muir 1999) and is typically performed in young animals to avoid discomfort, the potential loosening of screws and to restore full functional performance (Brinker 2013).

**Figure 1:**
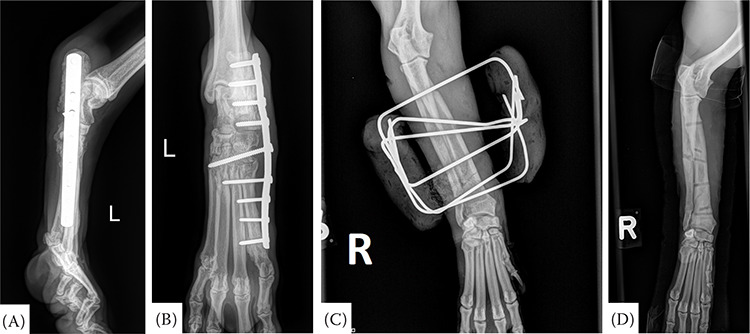
Absolute implant removal indication in two patients (A) Post-operative radiographic images of the left tarsus of a 3-year-old intact male Labrador Retriever after partial arthrodesis. Mediolateral view of the tarsus with visible osteomyelitic changes on the caudal surface of the calcaneus. (B) Dorsoplantar imaging of the tarsus of the same dog with visible osteomyelitic changes between the medial surface of the calcaneus and the proximal end of the plate. Implant removal is indicated. (C) Post-operative dorsopalmar views of the right antebrachium of a 5-year-old intact female mixed-breed dog after treatment of grade II open fracture of the radius and ulna with an external fixation device. (D) Radiographic dorsopalmar views of the same dog after implant removal. Implant removal is indicated to avoid bacterial translocation from the implant to the bone and to restore functional performance

Indications for the removal can be relative (not necessary) or absolute (when the removal is obligatory) (Figure 1A–D), and the decision-making process is influenced by a variety of factors. The aim of this study is to analyse arguments in favour and against implant removal in small animals, to compare it to the literature in human and equine medicine, and to investigate the existence of specific guidelines for small animal orthopaedics related to the removal of implants.

## ARGUMENTS IN FAVOUR OF IMPLANT REMOVAL

### Carcinogenesis and corrosion of implants

In human medicine, old implants used to be made of stainless steel, an alloy of chrome, nickel, and molybdenum. The risk of corrosion and oxidation of metals and the tendency of small particles that are potentially biologically active and can be shed into the surrounding tissue can lead to an inflammatory tissue reaction and consequently necrosis, granulation, and fibrous tissue. Additionally, concern over the development of cancers was a further motivator for the routine removal of hardware (Vos and Verhofstad 2013). These beliefs were contradicted by studies that failed to reveal any association between metal implants and the development of cancer (Grogan 1957). Since the nineties, corrosion and cancer formation is no longer considered to be an indication for the standard removal of implants (Vos and Verhofstad 2013).

On the one hand, it has been postulated that the presence of orthopaedic implants could disrupt osteogenic differentiation (Murphy et al. 1997; Rose et al. 2005) leading to the formation of osteosarcomas (Tang et al. 2008). There are studies suggesting that plate removal should be advised to owners to avoid the risk of osteosarcomas, as long-term inflammation could possibly create a tumorigenic environment. This could be promoted by the presence of non-medical material (i.e., vinyl chloride bands), stainless steel, or titanium implants, which have been proven to induce inflammation and have carcinogenic properties (Operkalski et al. 1987; Kirkpatrick et al. 2000; Isaka et al. 2021).

On the other hand, in small animal medicine, cancer formation is not considered to be an indication for implant removal, even if there are various case reports suggesting neoplastic phenomena possibly induced by orthopaedic implants (Harrison et al. 1976; Sinibaldi et al. 1976; Roe et al. 1996; Murphy et al. 1997; Marcellin-Little 1999; Boudrieau et al. 2005; Dunn et al. 2012; Burton et al. 2015; Arthur et al. 2016). Although fracture-related osteosarcomas in veterinary medicine have previously been reported, it is extremely rare, and implant removal to reduce the risk of osteosarcoma after fracture healing may not be warranted (Arthur et al. 2016).

### Bacterial infection

Infections in orthopaedic trauma patients is uncommon in human medicine and varies significantly depending on the location, severity of the injury and the type of fracture, from 1–2% in internal fractures up to 30% in open fractures (Trampuz and Zimmerli 2006). Common findings after infection are pain, delayed healing, infection, osteomyelitis, implant loosening, and loss of implant function (Langkamer and Ackroyd 1990; Khan et al. 2008; Ackling et al. 2018). Infections do not necessarily have to be an indication for implant removal, as some authors recommend stabilising the fracture in order to treat the infection and leaving the hardware in place until the fracture healing is completed. This strategy, in combination with local and systemic antibiotic therapy, has been proven to be a successful concept (Trampus and Widmer 2006; Rightmire et al. 2008; Berkes et al. 2010).

The type of fracture, type of internal fixation plate or material, and the better biocompatibility of titanium implants have all been shown to be decisive factors affecting the development of bacterial infections in human orthopaedics (Melcher et al. 1996; Eijer et al. 2001; Schlegel and Perren 2006; Stewart et al. 2015). Also, the efficiency of prophylactic antibiotics in open fractures has been confirmed to reduce the infection rate from 13.4% to 5.5%, suggesting that patients should be started on intravenous antibiotics within 3 h of injury and that antibiotics are an adjuvant to a thorough debridement and not a substitute (Gosselin et al. 2004). First-generation cephalosporines are significantly more effective than a combination of other antibiotic groups such as penicillins or aminoglycosides (Bergman 1982; Braun et al. 1987).

One of the most common causes for implant removal in small animal medicine is the development of bacterial infections and it has shown to be the most common cause for tibial plateau levelling osteotomy plate explantations (Thompson et al. 2011) with an incidence of 3% of surgically treated patients (McDougall et al. 2021). In a larger study, the second most common reason for hardware removal was bacterial infections, with an incidence of 21% (Emmerson and Muir 1999). The presence of microorganisms in explants appears to be a common finding in around 50% of small animals, with clinically relevant agents present in only 7.3% of cases. Older, overweight patients and patients with lung lesions were more prone to develop a bacterial infection (Pagel 2015; Slunsky et al. 2017). The presence of microorganisms on an implant does not necessarily lead to treatment complications, but once infection and its clinical signs are present, implant removal leads to better results and superior outcomes in dogs (Savicky et al. 2013).

According to equine medicine reports, the overall postoperative infection rate is 28%, with infections related to implant loosening being an inescapable justification for plate removal (Auer and Grainger 2015; Ruggles 2013). In horses, the main indication for implant removal is infection of the plate with an incidence of 35% of the total extracted plates (Donati et al. 2021).

Osteomyelitis is a difficult condition in veterinary medicine that is frequently caused by infection and requires debridement, antibiotic therapy, and implant removal when indicated (Figure 1A,B) (Gieling et al. 2019).

### Avascular necrosis

Bone atrophy has been used as an argument for implant removal in human orthopaedics (Kettunen et al. 2003). Implants have been found to decrease the strength of the underlying bone structure (Rosson et al. 1991a; Rosson et al. 1991b) and cortical atrophy and demineralisation have been described as a consequence (Rosson et al. 1991c). The type of plate might be decisive in the appearance of such side effects and previous biomechanical studies demonstrated the advantages of locking plates over conventional plates (Fulkerson et al. 2006). In contrast to this, recent studies proposed that locking plates in forearms in humans could lead to more bone atrophy due to long-term stiffness (Matsuura et al. 2017). These mentioned factors remain controversial and further studies are warranted.

In small animals, recent studies have shown the superiority of locking plates over conventional plates. Implant induced osteoporosis could be a more common complication in small breed dogs because the periosteum is compressed with the placement of the plate, leading to a reduction of bone mineral density, the thinning of the cortical bone, and an increase in the number of osteoclasts due to impaired periosteal blood flow (Muroi et al. 2021).

It has been proposed that due to these alterations in the bone structure and fragility, mainly caused by the screw holes after internal fixation, it is preferable to delay the plate removal in humans for at least 21 months or not remove them at all (Rosson et al. 1991c; Vos and Verhofstad 2013). All these aforementioned factors complicate the decision to remove internal plates.

### Implant failure

A clear indication for implant removal in human orthopaedics is implant failure, including breakage, angulation, and other mechanical issues. These complications occur in 6.3 to 8.5% of cases (Gilde et al. 2014; Woltz et al. 2016; Hulsmans et al. 2017).

The rate of implant failure lies between 0 and 21% in small animal orthopaedics, depending on the type of locking plate system, with the string of pearls (SOP) locking plates accounting for the highest incidence of failures (Hudson et al. 2009; Guerrero et al. 2014; Gilbert et al. 2015; Field et al. 2018).

**Figure 2:**
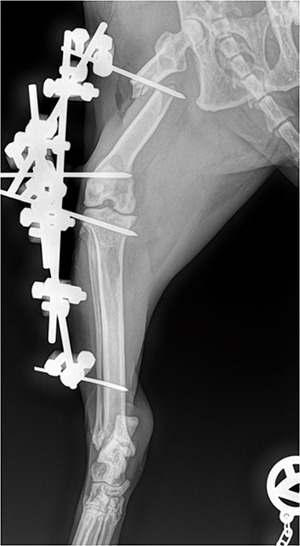
Ventrodorsal radiograph of the hip and right hind bones of a 13-year-old neutered male European shorthair cat with an implant associated fracture Disruption of the right stifle was stabilised with a transarticular external fixator device. Removal of this assembly is generally recommended 4 weeks after the initial surgery. As basic principles were not followed, a stress fracture associated with the proximal pin occurred 8 weeks after the first surgery

Plates utilised to bridge fractures rather than neutralise or compress them have proven to be a substantial risk factor in both human and small animal orthopaedics. Even though plate breakage is rare, further studies regarding the risk factors and pathogenesis are needed and basic principles in osteosynthesis must be followed (Figure 2) (Field et al. 2018; Batash et al. 2019).

### Angular deformations in growing patients

**Figure 3:**
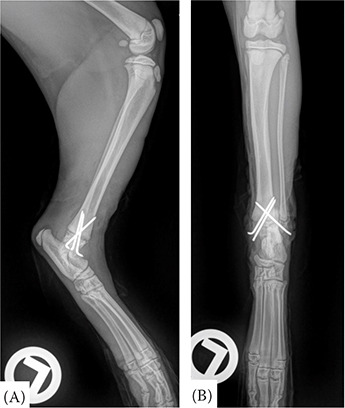
Radiographic imaging of the stifle joint, tibia, and tarsus of a 5-month-old female spayed European shorthair cat with a distal tibial epiphysiolysis Because the implant is placed in the epiphysial growth plate, but does not interfere with its growth, this can be considered a relative indication for implant extraction. (A) Mediolateral view. (B) Plantodorsal view

Implant removal in the growing skeleton has always been thought to be necessary in human orthopaedics to avoid angular deformations or interference with bone growth, but even this argument has become disputed and is no longer considered an unquestionable indication, provided that physeal growth is not restricted (Figure 3A,B) (Loder and Feinberg 2006; Morshed et al. 2007; Raney et al. 2008; Gorter et al. 2011).

**Figure 4:**
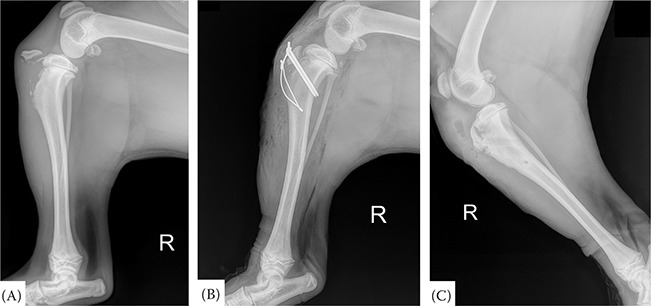
Mediolateral radiographic views of the right stifle joint, tibia, and tarsus of a 6-month-old intact male English Bulldog after tibial apophysiolysis Kirschner-wires were placed through the epiphysial plate, thereby blocking further growth. To avoid growth interference, the implant should be removed as soon as possible. (A) Pre-surgical view. (B) Post-surgical view. (C) Post-explantation view

The timely surgical treatment of fractures in young small animals with fractures or trauma in the physeal area is necessary to avoid alterations in bone growth which could result in the shortening or distortion of the limb (Figure 4A–C).

To avoid complications, plates should be minimal in size and removed between 3 and 5 weeks depending on the age and circumstances (Brinker et al. 1990).

Following the same principles, implant removal is proposed in growing equine patients to prevent developmental abnormalities, such as angular limb deformities. This could assist horses in achieving peak athletic performance (Richardson 2008) and is one of the main reasons for implant removal in young equine patients (Donati et al. 2021).

### Other indications for implant removal in small animals

Other indications for removal proposed by Brinker (2013) in their guidelines for bone internal fixation include:

#### THERMAL CONDUCTION

**Figure 5:**
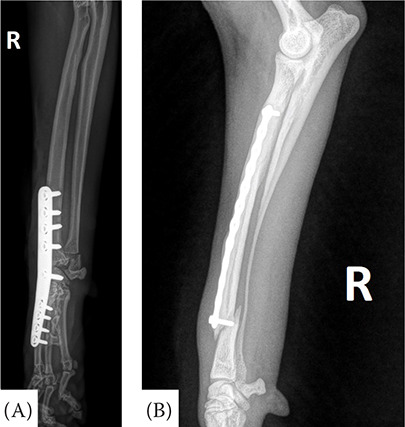
Other indications for implant removal (A) Post-operative mediolateral views of the right forearm and carpus of a 4-year-old spayed female European shorthair cat after carpal hyperextension. A relatively thick bone plate only covered by thin subcutaneous tissue and skin causes discomfort and impairment of the functional performance. (B) Mediolateral radiograph of the elbow, forearm, and carpus of a 2-year-old intact male Spitz with a radius and ulna fracture. A fracture occurred near to the distal end of the plate twelve months after the initial surgery. The concentration of forces and the consequent fracture at this location may have been averted if the implant had been removed earlier

Mostly described in fractures with a thin layer of soft tissue covering the implant. The external temperature can cause discomfort due to the changing plate temperature, as seen in dogs who limp when they are outside in the cold, but resume normal function once they are inside (Figure 5A).

#### SOFT TISSUE IRRITATION

Lick granulomas can occur when the end of a plate is close to a joint and is only covered by subcutaneous tissue and skin. Implant removal should be performed once the fracture has healed.

#### STRESS PROTECTION

Since plates are more rigid than bones, they hinder the bone from responding to normal physiological cues, causing changes in its architecture and density. There is evidence that this phenomenon is triggered by interference with the local vasculature.

#### ACCOMPLISHMENT OF OBJECTIVE

As the fracture heals, the plate serves no further purpose and may impair full functional performance, especially in field and racing animals. Many screws loosen once the fracture has healed. Even if a reasonably long portion of bone extends beyond the last screw in the plate, a fracture may form at this point due to an elastic mismatch between the bone and the plate, even if the original fracture has healed (Figure 5B).

## ARGUMENTS AGAINST IMPLANT REMOVAL AND COMPLICATIONS ASSOCIATED WITH IT

Studies in human patients after implant removal have shown up to 91% improvement in symptomatic patients. However, implant removal is not always easy and does not necessarily come without risks. Potential rates of complications range from 3% to 40% in human medicine (Richards et al. 1992; Sanderson et al. 1992; Evers et al. 2004; Raney et al. 2008; Berkes et al. 2010; Raja et al. 2012) and include nerve lesions, wound healing disorders, haemorrhage, infections, refractures, or the incomplete removal of hardware (Langkamer and Ackroyd 1990; Muller-Farber 2003; Reith et al. 2015).

**Figure 6:**
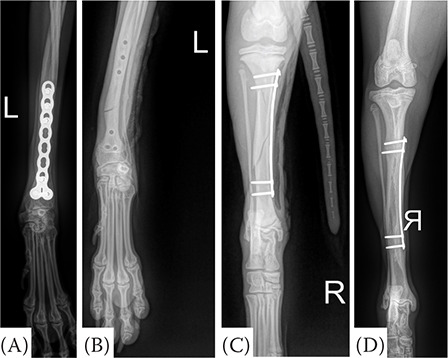
Factors against implant removal (A) Dorsopalmar radiographic post-surgical views of the left forearm of a 1-year-old intact male Italian Greyhound after a radius and ulna fracture. Implant extraction was performed 8 weeks after the first surgery. (B) Dorsopalmar imagining the same patient 4 days after the early implant extraction, with a refractured distal radius. (C) Post-surgical dorsoplantar imaging of the right stifle, tibia and tarsus of a 4-month-old male Prague Ratter after a tibial and fibular fracture. (D) Dorsoplantar radiological views of the same Prague Ratter months after the surgery. Visible bone overgrowth that could lead to intraoperative complications when removing the implant, such as cortical bone trauma and risk of refracture. Implant removal not recommended

More surgical complications must be expected when implants, particularly titanium implants, are left in place for an extended period of time (DGU 2018). Arguments against implant removal are also influenced by the location, type and material of the hardware. Implants are sometimes only identified with difficulty, and removal can prove challenging due to bone overgrowth (Figure 6C,D), stripping of the screw head, and implant breakage, meaning that procedures that were minimally invasive for implantation might become maximally invasive for explantation (Brinker 2013; Vos and Verhofstad 2013). Decision-making can also be influenced by socioeconomical factors such as extra costs or an additional recovery period (Vos and Verhofstad 2013) (see Table 1).

**Table 1:** Summary of the arguments in favour and against hardware removal in human, equine and small animal medicine

In favour of removal	Against removal
Carcinogenesis and corrosion of implants	Risks derived from additional surgery
Bacterial infection	Risk of refracture after removal
Avascular necrosis	Nerve lesions or haemorrhage (Figure 7)
Breakage of internal plates	Incomplete hardware removal
Angular deformations in growing patients	Infection
Other indications: - Thermal conduction - Soft tissue irritation - Stress protection - Accomplishment of objective	Socioeconomical factors such as: - Costs - Recovery period

**Figure 7:**
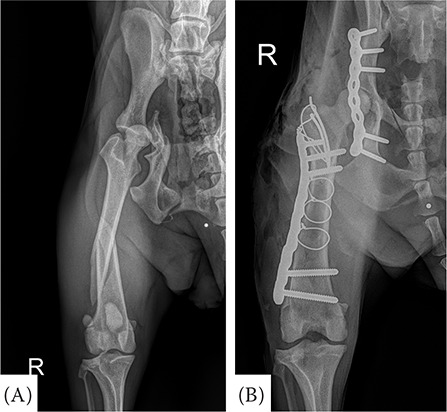
X-rays of the right hip, femur and stifle joint of a 3-year-old female mixed breed dog after a femur and acetabular fracture before (A) and after (B) surgical treatment Implant extraction is not recommended in this case due to potential intraoperative complications, such as nerve damage and haemorrhage

Refractures in small animals may arise as a result of early implant removal (Figure 6A,B) or due to the poor anatomical correction of the fracture when basic principles are not followed. It may be difficult to determine if a plate can be removed since some fractures appear to have healed radiographically, but the bone diameter at the fracture site has decreased (Figure 1A,B) (Brinker 2013).

In equine medicine, refractures and persistent infections were the most common complications after hardware removal. With the limitation of much smaller study samples, an overall complication rate of 12.5% was discovered; however, these results are unlikely to be comparable with studies in human patients, as a mild discomfort in a pasture-sound horse after implant removal could easily be interpreted as an excellent outcome (Donati et al. 2021). Refractures were substantially more prevalent after plate removal in weight-bearing long bones and elderly patients (Bischofberger et al. 2009; Stewart et al. 2015; Donati et al. 2021).

## DISCUSSION

Although the complex process of fracture healing is similar in human and veterinary medicine, animal orthopaedics can prove more challenging due to owner incompliance, a lack of cooperative animal behaviour, and difficulty in applying adequate confinement and activity restriction during the healing process. In some cases, the lack of implants particularly designed for animal anatomy complicates the process even further.

Although there are several advantages to implant removal, such as functional improvement and pain relief, the surgical procedure can be very challenging and may result in complications such as neurovascular injuries or refractures (Jain et al. 2019). Indications for removal can be relative (not necessary) or absolute (when removal is obligatory), and the decision-making process is influenced by a variety of factors, i.e., surgery-related factors (differences between countries, hospital protocols), implant-related factors (type of material), patient-related factors (age, implant location, human or animal), or socioeconomical factors (insurance coverage, financial restraints) (Vos and Verhofstad 2013).

Indications for hardware removal in humans are diverse and hardly supported by literature, as most publications are retrospective studies and case reports (Vos and Verhofstad 2013). Implant removal in human patients without medical indication (elective) is controversially discussed and there is insufficient evidence to support implant removal in extremities (Prediger et al. 2020). These issues, as well as the lack of a consensus not only in human, but also in veterinary medicine, could easily lead to implant failure or removal complications. Therefore, more specific, clearer guidelines for the removal of implants not only in human medicine, but in veterinary medicine, are needed.

Although several studies have linked implants to carcinogenesis (Harrison et al. 1976; Sinibaldi et al. 1976; Roe et al. 1996; Marcellin-Little 1999; Murphy et al. 1997; Dunn et al. 2012; Boudrieau et al. 2005; Burton et al. 2015; Arthur et al. 2016), corrosion and cancer formation are extremely unusual occurrences which are no longer accepted as reasons for implant removal in both human and small animal medicine (Grogan 1957; Arthur et al. 2016).

Even bacterial infections do not appear to be a clear indication for implant removal in humans, as some authors recommend fracture stabilisation, as well as local and systemic antibiotic administration for treatment of infections (Trampuz and Widmer 2006; Rightmire et al. 2008; Berkes et al. 2010). Studies have shown that the presence of microorganisms on implants taken from small animals does not necessarily lead to healing complications, because only a small proportion of those pathogens are clinically relevant. However, when symptoms do occur, the removal of the implants is recommended (Savicky et al. 2013; Slunsky et al. 2017). Different approaches to reduce the infection incidence have been developed, such as coating implants with antibiotics and covalently attaching antimicrobial molecules onto the implant surface. These alternatives can disrupt the metabolic machinery of the microbes or prevent bacterial adhesion to the implant (Ketonis et al. 2012).

Other symptomatic processes like avascular necrosis or breakage of internal plates are absolute indications for implant removal (Brinker 2013; DGU 2018).

Guidelines for implant removal in human medicine have been created by the German Society for trauma surgery (DGU 2018). These guidelines were written in German, are periodically updated, and based on scientifically proven study results and therapeutic consensus. They, therefore, offer a very quick overview regarding indications, procedures, and potential complications that can be found in different cases. They are drawn up in brief and are not intended to be a substitute for textbooks or operating instructions, and furthermore do not address some important topics like antibiotic therapy or thrombotic prophylaxis.

To the authors’ knowledge, there are currently no internationally accepted consensus statements concerning this topic.

In small animal medicine, as well as in equine medicine, detailed guidelines have not yet been created, but some indications for the removal of implants such as loss of functionality, thermal conduction, irritation, infection, stress protection or the accomplishment of objective have been proposed (Brinker 2013).

Over the last decades, veterinary medicine has achieved great improvement in the care of companion animals. Although the basic principles of orthopaedics are the same, irrespective of the species, surgeons treating cats should be aware of the important differences that exist with respect to dogs as their anatomy and function as well as management of fractures and orthopaedic conditions might be different.

The indoor/outdoor lifestyle of domestic cats, anatomical differences, the cat’s uncooperative nature and limitations to apply activity restrictions complicate the fracture healing process further. There is a widespread belief that repairing feline long bones is straightforward and free of difficulties. This belief may have originated as a result of cats’ superior ability to adjust for reduced function (Piras 2009). All these factors can influence the surgeon’s decision making process to remove plates in feline patients and should be taken in account when creating guidelines.

Potential complications in human patients undergoing an implant extraction include haemorrhages, nerve lesions, post-explantation infections, and refractures (Vos and Verhofstad 2013; DGU 2018). Studies conducted in small animal medicine also describe these complications as possible events after an implant extraction, but generally, all these studies were performed in patients that underwent implant extraction for a major reason, i.e., instability, infection, soft tissue irritation or discomfort (Emmerson and Muir 1999).

To our knowledge, there are currently no studies analysing complications in healthy patients without healing complications that underwent an elective implant extraction procedure. We believe that the potential complications might vary between patients with a relative and absolute implant extraction, as the presence of a bacterial infections or damaged soft tissue due to a broken plate could also affect the overall healing process. Healing complications can influence the complexity of implant extraction and the presence of post-explantation infections, haemorrhages, nerve injury, or refractures. This should be also considered when creating new guidelines.

Studies in horses showed that the fracture site and age of patients influences the overall outcome of the healing process. Younger horses with a lower weight and more efficient bone remodelling had better outcomes. Bacterial infections (especially after an open fracture) and fractures in weight bearing bones considerably affected the presence of complications (Donati et al. 2021). This could be an example of how elective or absolute procedures can affect the overall outcome.

Bio-absorbable implants have been successfully used in non-weight-bearing fractures in human and small animal medicine, with the advantage of avoiding the removal of metallic implants (Bos et al. 1989). These implants are an excellent alternative, but they require lengthier operation times and high-quality research to fully understand their potential (Gareb et al. 2021).

## CONCLUSIONS

In small animal medicine, as well as in human and equine orthopaedics, indications for implant removal after fracture healing are still under debate and an international consensus is presently lacking. Large differences in opinions, practices between surgeons, countries, patients, species, location, and type of implants exist.

In human and veterinary medicine, only few clear recommendations for implant removal currently exist and further studies and guidelines are needed. Future consensus statements should not only take factors like age, weight, type and site of fracture in account, but the differences between species, as the anatomy, biomechanics and activity restriction strategies might be different. Even if there are several supposed benefits of implant removal, the surgical procedure can be very challenging and may lead to complications, thereby worsening the situation. Protocols should be adapted based on recent solid scientific information and should consider evolving strategies like the antibiotic or antimicrobial coating of implants to prevent infections or the use of other devices, such as bio-absorbable implants, as an alternative in some specific cases, with the great advantage of avoiding implant removal surgery and its possible complications.
